# The potential therapeutic role of curcumin in osteoporosis treatment: based on multiple signaling pathways

**DOI:** 10.3389/fphar.2024.1446536

**Published:** 2024-08-08

**Authors:** Keyu Wang

**Affiliations:** College of Bioscience and Biotechnology, Hunan Agricultural University, Changsha, China

**Keywords:** curcumin, osteoporosis, herbal medicine, mechanism, pharmacological effect, pathway

## Abstract

Osteoporosis is a common chronic metabolic bone disease caused by disturbances in normal bone metabolism and an imbalance between osteoblasts and osteoclasts. Osteoporosis is characterized by a decrease in bone mass and bone density, leading to increased bone fragility. Osteoporosis is usually treated with medications and surgical methods, but these methods often produce certain side effects. Therefore, the use of traditional herbal ingredients for the treatment of osteoporosis has become a focus of attention and a hot topic in recent years. Curcumin, widely distributed among herbs such as turmeric, tulip, and curcuma longa, contains phenolic, terpenoid, and flavonoid components. Modern pharmacological studies have confirmed that curcumin has a variety of functions including antioxidant and anti-inflammatory properties. In addition, curcumin positively regulates the differentiation and promotes the proliferation of osteoblasts, which play a crucial role in bone formation. Multiple studies have shown that curcumin is effective in the treatment of osteoporosis as it interacts with a variety of signaling pathway targets, thereby interfering with the formation of osteoblasts and osteoclasts and regulating the development of osteoporosis. This review summarized the key signaling pathways and their mechanisms of action of curcumin in the prevention and treatment of osteoporosis and analyzed their characteristics and their relationship with osteoporosis and curcumin. This not only proves the medicinal value of curcumin as a traditional herbal ingredient but also further elucidates the molecular mechanism of curcumin’s anti-osteoporosis effect, providing new perspectives for the prevention and treatment of osteoporosis through multiple pathways.

## 1 Introduction

As the aging population grows, osteoporosis (OP) has emerged as one of the top three major chronic diseases in China (Yu and Xia, 2019). OP is a disease in which bone mass is reduced, microstructure deteriorates and fragility fractures occur. The main reason is the upregulation of osteoclasts, resulting in an imbalance between osteoblasts and osteoclasts, which are the basic cells for bone growth and maintenance because they form bone tissue, and an imbalance between the two can lead to OP (Visconti et al., 2019). Research findings have shown that in the maintenance of the bone metabolic balance, the critical stem cells are BMSC (Wang et al., 2016). Furthermore, the osteogenic conversion of bone marrow mesenchymal stromal cells (BMSCs) is a primary provider of osteoblast precursor cells (Wilkesmann et al., 2020). Osteoblasts are fundamental cells that produce bone, controlling mineralization, and driving bone development (Florencio-Silva et al., 2015). Therefore, a sufficient source of osteoblasts is the key to ensuring bone reconstruction and maintaining bone metabolic balance. OP is often seen to fall in either primary or secondary category and it affects people of all ethnic backgrounds, as well as many older men and women (Romagnoli et al., 2011). Preventing OP calls for reaching a normal peak bone mass, but this requires an individual to nourish their body with a healthy diet comprising calcium, vitamin D in plenty, regular menstrual cycles, and a comprehensive physical workout schedule (Weaver et al., 2016). Menopause triggers significant hormonal fluctuations and localized oxidative inflammation predominantly in women, disrupting the delicate balance maintained by osteoclasts and osteoblasts. This disruption accelerates the process of bone degeneration, ultimately contributing to the development of OP, a condition characterized by weakened bones and increased fracture risk (Baastrup, 2016).

Since ancient times, Chinese herbal medicine has constituted an indispensable and fundamental part of the medical field, carrying with it profound traditional wisdom (Yuan H. et al., 2016). The advancement of modern science and technology has greatly facilitated the in-depth study of Chinese herbal medicine, enabling scientists to accurately analyze its chemical composition and elucidate its complex pharmacological mechanisms (Lihong, 2019). This has resulted in the establishment of a more solid scientific foundation for the application of traditional Chinese herbal medicine. Currently, Chinese herbal medicine is gradually becoming a bridge and intersection point connecting traditional and modern, Eastern and Western medicine (Scheid, 2002). Its wide variety and different efficacy are not only widely used globally for the treatment of all kinds of diseases, but also become a vivid embodiment of the concept of mankind’s pursuit of a harmonious symbiosis with nature. From the long heritage of the ancient classic Shennong’s Classic of Materia Medica to the continuous exploration of modern scientific research, Chinese herbs have consistently played a pivotal role in the history of human medical practice, and continue to contribute to human health and wellbeing in a unique way (Liu et al., 2018). Over time, they have developed a comprehensive theoretical framework and a set of sophisticated practical techniques, which have been passed down from generation to generation through the accumulated wisdom of countless medical practitioners. The essence of Chinese herbal medicine lies in the uniqueness of its “four qi and five flavors” concept, which reveals the subtle regulating effects of the four qualities of cold, heat, warmth, and coolness, as well as the five flavors of acidity, bitterness, sweetness, pungency, and saltiness on the balance of the human body’s internal environment (Zhaoguo et al., 2021). This concept enables the comprehensive goals of disease prevention, treatment, and healthcare to be achieved.

In recent times, turmeric has garnered widespread attention for its potent anti-inflammatory, pain-relieving, and blood circulation-enhancing properties, as documented in reference (Yuandani et al., 2021). Through meticulous clinical research, scientists have successfully identified and isolated key constituents from the turmeric root, including polysaccharides and the bioactive compound curcumin (CUR), as noted in reference (Jiang T. et al., 2021). The discovery that turmeric extracts are capable of inducing both vasoconstriction and vasodilation has significantly expanded our comprehension of turmeric’s multifaceted therapeutic mechanisms. This insight also offers a crucial perspective for the development of medicinal spices rich in curcumin, potentially revolutionizing the field of herbal medicine.

CUR is a bioactive compound extracted from the root of the turmeric plant and has excellent antioxidant and anti-inflammatory properties due to its unique phenolic structure. Several studies have confirmed its effectiveness in combating intracellular reactive oxygen species (ROS) and scavenging free radicals, thereby reducing oxidative stress and enhancing the immune system (Iddir et al., 2020). Therefore, CUR has been widely used in the treatment of cancer, cardiovascular diseases, osteoporosis, etc. The special molecular composition of CUR endows it with unique chemical properties, and the morphological equilibrium of its heptadienone molecules in different pH environments is crucial for its antioxidant and physicochemical properties (Stanić, 2017; Luca et al., 2020; Slika and Patra, 2020).

In addition, CUR has been reported to protect against oxidative damage and promote osteoblast differentiation by attenuating the inhibition of Wnt/βcatenin signaling (Manandhar et al., 2020). CUR can not only reduce the oxidative state of mitochondria but also improve the mitochondrial membrane potential and improve the oxidative stress-induced apoptosis of osteoblasts (Inchingolo et al., 2022). Therefore, CUR promotes the osteogenic differentiation and bone formation of BMSC by interfering with BMSC, osteoblasts, and osteoclasts. In addition, it promotes the growth and specialization of osteoblasts while inhibiting the proliferation and differentiation of osteoclasts, ultimately increasing bone density and improving the microstructure of bone trabeculae. For example, AHMED et al. found that adding CUR to a petri dish helped enhance the osteogenic differentiation of mouse BMSC (Ahmed et al., 2019). Not only that, but CUR can also protect osteoblast function by anti-oxidative stress. According to Li et al., Cur preconditioning reduced the apoptosis of osteoblasts and maintained their differentiation function by eliminating the inhibitory effect of ROS on the GSK3β-Nrf2 signaling pathway (Li et al., 2020a).

**FIGURE 1:**
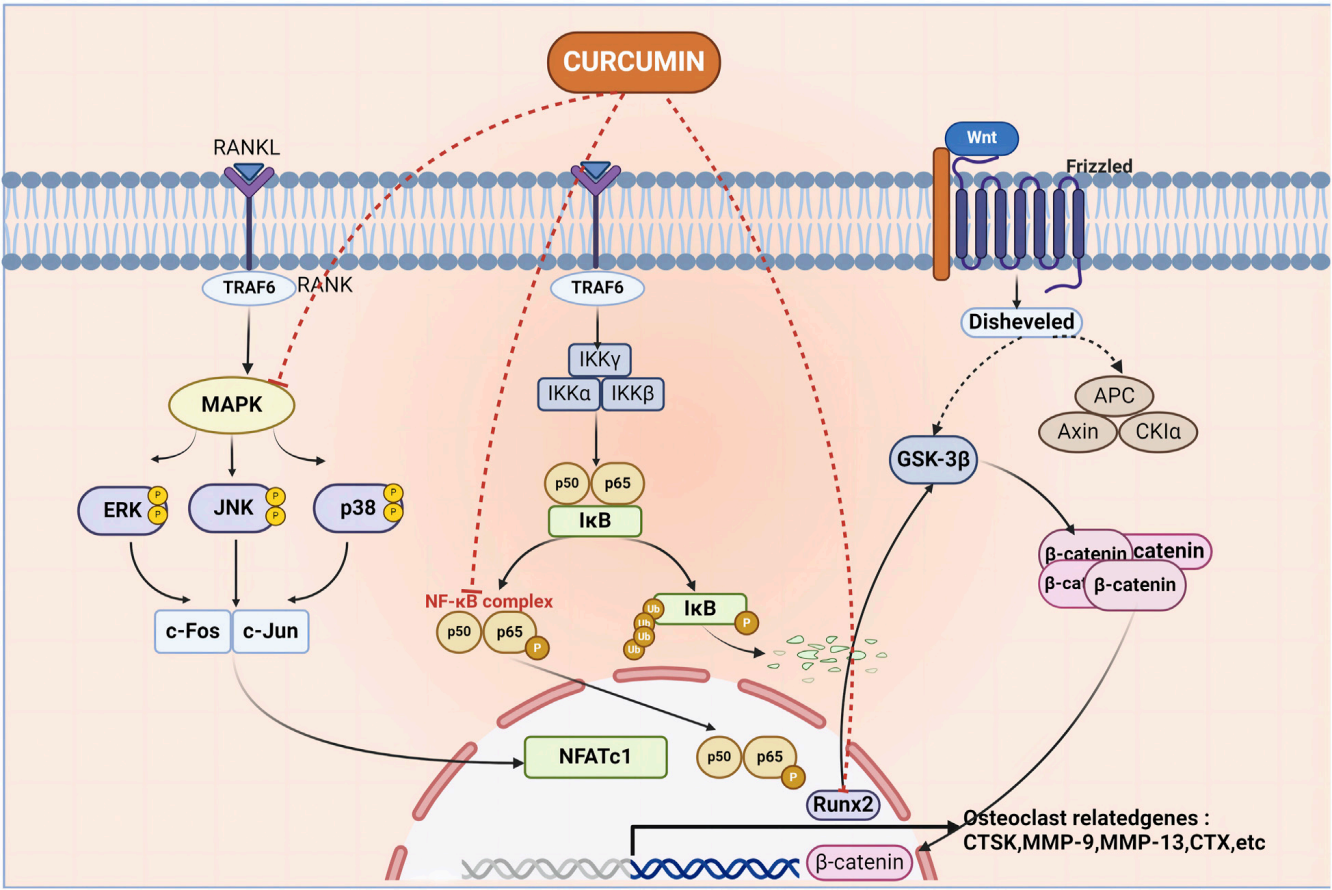
Curcumin acts on multiple pathways to inhibit the formation of osteoclasts, thus achieving the purpose of treating osteoporosis. Curcumin mediates the occurrence of osteoporosis by acting on MAPK, NF-κB, Wnt signaling pathway, MAPK, nuclear factor kappa-B complex, and Runt-related transcription factor 2 molecules.

Mitogen-activated protein kinase (MAPK) can be switched on by a variety of extracellular stimuli and consists of a conserved tertiary kinase pattern (Bharti et al., 2021). This particular pathway helps regulate various bodily processes, including cell growth, stress response, and inflammation. It is a part of the signal transmission network in cells, regulating gene expression and cytoplasmic activity (Olivares-Bañuelos et al., 2019). In addition, there are specific pathways in the MAPK signaling pathway, such as the ERK1/2 pathway, the c-Jun N-terminal kinase (JNK) pathway, the P38 pathway, and the Extracellular signal-regulated kinase 5 (ERK5) pathway, which have been associated with diseases such as OP and bone formation (Rodríguez-Carballo et al., 2016). Innate immune cells and inflammatory T cells are regulated by nuclear factor-kappa B (NF-κB) through its ability to modulate gene expression. The function of NF-κB is important in the context of different pathogens and infections as it ensures an adequate immune response (Liu et al., 2017). Two Rel family proteins (p50/p65) make up the inactive form which is normally bound to inhibitory proteins (i‐κB) in the cytoplasm; the liberation of NF‐κB p65/p50 dimerization allows for its transportation into the nucleus. Upon reaching the nucleus, the p65/p50 dimer binds to certain DNA sequences and starts transcription for target genes that are important in different cellular activities such as cell growth (Wang et al., 2015). In a lot of illnesses, NF-κB is highly stimulated in the inflammation zones; this results in the production of pro-inflammatory mediators including cyclooxygenase-2 (COX-2) among others (Al-Harbi et al., 2016). Hence NF-κB has emerged as a target for the progression of anti-inflammatory and anti-cancer drugs. The study of NF-κB and its signaling pathway becomes unavoidable in appreciating its role in OP and devising ways to regulate its functioning efficiently. One of the physiological processes that the pathway for PI3K-AKT is associated with include obesity, diabetes, and cancer. Given its importance and potential for therapeutic intervention, it remains a fascinating and important area of research. Signaling cascades mediated by Phosphoinositide 3-Kinase (PI3K) and Protein kinase B (Akt) regulate key cellular activities and influence tissue-specific functions in adipose tissue, skeletal muscle, liver, brain, and pancreas (Savova et al., 2023). Class I PI3K regulates the production of the vital phosphatidylinositol second messenger that is essential for maintaining a balance in the body, it also contributes to the development of many human illnesses, such as cancer and metabolic ailments (Fruman et al., 2017). This process is initiated by the initiation of tyrosine kinase or G protein-coupled receptors, which leads to the generation of Phosphatidylinositol (Florencio-Silva et al., 2015; Wang et al., 2016; Wilkesmann et al., 2020) trisphosphate (PIP3). This PIP3 further triggers downstream effectors resulting in the amplification of the signal cascade (Oeckinghaus and Ghosh, 2009; Fruman et al., 2017). These signaling pathways have their characteristic targets for OP, and CUR can act on these targets to inhibit or activate the signaling pathway to achieve the purpose of treating OP (Figure 1).

## 2 Curcumin

Throughout China’s long history, herbs have consistently held a prominent position in the treatment of human diseases and the maintenance of health. This reflects the profound depth and rich heritage of traditional Chinese medicine (Unschuld, 2018). Chinese herbs, a magnificent cultural legacy, have been used for thousands of years, not only embodying the wisdom and vitality of the ancients but also representing a precious gift from nature. They encompass a vast array of subjects, including plants, animals, and minerals (Hageneder, 2020). Each type of Chinese herbal medicine is a unique chemical treasure trove in nature, containing a variety of pharmacological components such as flavonoids, alkaloids, polysaccharides, volatile oils, etc (Kuzmishyna, 2024). These components exhibit multiple biological activities, including anti-inflammatory, anti-bacterial, anti-viral, enhancement of immunity, and optimization of microcirculation in the body. This highlights the infinite charms of Chinese herbal medicine.

Chinese medicine theory profoundly recognizes that the human body is a complex and sophisticated organic system and that the occurrence of diseases is often a reflection of the imbalance of yin and yang in the body and the dysfunction of the internal organs. Therefore, Chinese herbal medicine treatment upholds the principle of “evidence-based treatment”, i.e., based on the patient’s specific condition, physical differences, age and gender characteristics, a careful and detailed consideration, carefully formulated drug combinations, and strive to achieve both symptomatic and curative effects (Hoover, 2021). In addition, the scope of application of Chinese herbs goes far beyond drug treatment, they are also cleverly integrated into food therapy, medicinal meals healthcare products, and other areas of life, becoming an indispensable part of the pursuit of health and enjoyment of life (Hong et al., 2022). In recent years, with the rapid development of science and technology, there has been an explosion of research on the isolation and development of biologically active components from Chinese herbal medicines, which have attracted much attention because of their remarkable pharmacological properties. What is even more gratifying is that the influence of Chinese herbs and their preparations has crossed national borders, and they are not only trusted in China but are also gaining recognition and popularity in Europe and North America as complementary therapeutic means (Rausch et al., 2024). With the help of advanced extraction, separation, and identification technologies, we have been able to explore the pharmacological mechanisms of Chinese herbs and reveal their scientific mysteries, contributing indelibly to the cause of human health and medical progress.

Among them, turmeric is particularly widely used in ancient medicinal formulas, and its medicinal value has been fully reflected in traditional Chinese medicine formulas. For example, turmeric powder paired with other herbs can effectively treat intractable cardiac pain as documented in Sheng Ji Gong Lu (Liu, 2024). This is due to the efficacy of turmeric in activating blood circulation and removing blood stasis, thus relieving heart pain. It is recorded in ancient books that turmeric is used in combination with Citrus aurantium and cinnamon bark to relieve stomach pain due to liver depression and stagnation of qi (McCaskill, 2021). This shows that the combination has unique efficacy in dispersing the liver and regulating qi, moving qi, and relieving pain.

**FIGURE 2:**
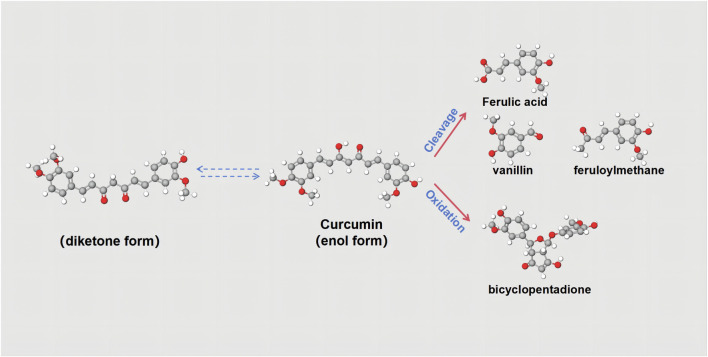
Chemical structures of curcuminoids. Curcumin has keto-enol tautomers, and the two can be converted to each other. Curcumin can be split into Ferulic acid, vanillin, and feruloyl methane. It can also be oxidized to bicyclopentadione.

CUR, an extracted component of herbal medicine, is also a highly versatile natural polyphenolic compound that can be isolated from the rhizomes of plants in the *Zingaceae* and Araceae families, such as Curcuma (*C. zedoaria (Berg.) Rosc.*), tulip (*Curcuma aromatica Salisb.*) and others (Jabczyk et al., 2021). In addition, CUR boasts a rich history of widespread consumption as a dietary spice and food coloring, and it is increasingly garnering attention for its diverse pharmacological benefits, primarily its anti-inflammatory and antioxidant properties (Esatbeyoglu et al., 2012a). Its molecular formula is C_21_H_20_O_6_. In varying pH chemical environments, CUR possesses seven carbon atoms on its carbon chain and features two keto groups that can undergo keto-enol tautomerism. Consequently, CUR’s chemical structure is not stable at the physiological pH value (Stanić, 2017). CUR is almost completely insoluble in water, but soluble in organic solvents such as acetone and ethanol, and is fairly stable in the acidic pH of the stomach. From a chemical point of view, the molecule has two similar aromatic ring symmetrys and has conjugated double bonds as effective electron donors to hinder the formation of ROS (Parcheta et al., 2021). Therefore, the main function of CUR is reflected in its functional groups: phenolic groups and diketone structures. These two active functional groups mediate the hydrogen supply reaction of CUR, the Michael addition reaction, and a series of hydrolytic and enzymatic reactions (Figure 2) (Esatbeyoglu et al., 2012b). Despite its therapeutic potential, curcumin’s swift metabolism leads to poor oral bioavailability. Following ingestion, the majority is expelled through bile and feces, with a substantial portion (40%–85%) traversing the gastrointestinal tract unaltered, despite the presence of certain gut microbiota. To enhance its bioavailability, CUR can be co-administered with bromelain (Joshi et al., 2023). Additionally, researchers have proposed an innovative nanotechnology strategy to precisely overcome existing challenges and significantly advance the in-depth study of CUR in both *in vitro* experiments and *in vivo* applications (Li and Kataoka, 2020). This strategy exploits the distinctive benefits of nanoparticles, encompassing micelles, liposomes, and nanogels, to markedly enhance the efficacy of CUR through two fundamental mechanisms (Jacob et al., 2024). Primarily, by enhancing solubility, it effectively facilitates the dissolution and distribution of CUR in living organisms. Secondly, by prolonging the blood circulation time and ingeniously blocking unwanted metabolic pathways, it significantly improves the bioavailability of CUR. The second strategy is to significantly enhance the bioavailability of CUR by extending the blood circulation time and subtly blocking unnecessary metabolic pathways. The researchers also found that curcumin’s effectiveness may indeed vary among different populations (Salehi et al., 2019). The study indicates that women may absorb curcumin more effectively than men. Based on area under the plasma concentration-time curve comparisons, the bioavailability of micronized CUR was several times higher in women than in men (the exact value was five times higher in women than in men). This suggests that women may derive higher biological effects from CUR at the same dose. Similarly, for micellar curcumin, bioavailability was significantly higher in females than in males (114 times higher in females than in males).

Derived from natural plants, CUR is a ubiquitous bioactive supplement widely used in the treatment of chronic diseases, including cardiovascular diseases, diabetes, malignancies and osteoarthritis (Table 1). CUR counteracts inflammatory mediators and has the ability to neutralize reactive oxygen species in the body. In addition, it enhances the activity of enzymes such as superoxide dismutase and glutathione peroxidase, thereby strengthening the body’s antioxidant defenses (Sharifi-Rad et al., 2020). CUR is a natural compound that modulates a variety of signaling pathways and possesses both anti-inflammatory and antioxidant properties, and therefore has a positive impact on bone health (Antolin et al., 2024). Recent studies have shown that CUR can influence bone formation by regulating the distinction between osteoblasts (Blair et al., 2017). It promotes the proliferation of osteoblasts and increases the expression of important genes critical for bone formation, including alkaline phosphatase, osteocalcin (OCN), Runt-related transcription factor 2 (Runx2) and various other markers (Yang et al., 2023a). Furthermore, in studies investigating the efficacy of curcumin in the treatment of osteoporosis, researchers have used *in vivo* experiments to determine the optimal mode of administration and dosage due to the low bioavailability of curcumin. For example, in a related study conducted by Folwarczna et al. in an oestrogen-deficient rat model, it was observed that oral administration of 10 mg/kg/d for 4 weeks did not improve osteoporosis symptoms (Folwarczna, 2013). In contrast, other researchers increased the oral dose of curcumin to 110 mg/kg/d and showed that curcumin significantly enhanced oestrogen-deficiency-induced bone tissue morphology and markedly increased the number of osteoblasts in ovariectomized rats (Jiang Q. et al., 2021). These findings will be summarized in a subsequent review. It can therefore be concluded that the optimal mode of administration of curcumin is daily oral administration via feed and that lower doses may not achieve optimal therapeutic effects. Previous studies have found that the optimal therapeutic dose of curcumin is 110 mg/kg/d. Some findings related to potential side effects or adverse reactions associated with curcumin for the treatment of osteoporosis have emerged from recent studies and reports (Ghahfarrokhi et al., 2023). However, it is important to note that these findings may not be specific to the treatment of osteoporosis, but rather the general application of curcumin as a drug or supplement. For example, curcumin has been shown to potentially cause gastrointestinal disturbances, liver and kidney damage, and an increased risk of bleeding (Liu et al., 2022).

**TABLE 1:** Function and application of CUR.

Function	General medical/clinical research applications	Ref.
Antitumous effect	Curcumin treats cancer by targeting cell signaling pathways by regulating cytokines, enzymes, transcription factors, etc	Giordano and Tommonaro (2019)
Anti-inflammatory action	Curcumin can treat inflammation-related diseases such as inflammatory bowel disease, psoriasis, atherosclerosis, and OP	Peng et al. (2021a)
Antihyperlipidemic effect	For the treatment of diabetes, non-alcoholic liver disease, obesity, etc	de Sousa Guardiano Reis et al. (2022)
Antioxidant	Curcumin blocks the production of free radicals and is used in anti-aging products	Lima et al. (2011)
Antibiosis	Curcumin has an inhibitory effect on *Escherichia coli*, *Bacillus subtilis*, *Staphylococcus aureus*, *Bacillus* cereus and other foodborne pathogens and putrefactive bacteria	Gunes et al. (2016)
Antiischemic activity	Curcumin applied to laboratory rodents prevents edema and maintains the integrity of the blood-brain barrier	Li et al. (2016)
Antiviral	Curcumin or its derivatives can inhibit the gene expression and replication of virus and degrade the ubiquitin-proteasome system	Si et al. (2007)
Neuroprotective activity	Curcumin significantly improved memory in Alzheimer’s mice	Pan et al. (2008)
Anti-osteoporosis	Curcumin prevents diabetic osteoporosis by promoting osteogenesis and angiogenesis coupling through NF-κB signaling	Fan et al. (2022)
Anti-osteoporosis	Curcumin alleviates glucocorticoid-induced osteoporosis by protecting osteoblasts from apoptosis *in vivo* and *in vitro*	Chen et al. (2016a)

CUR, as an effective Chinese herbal medicine, has little adverse reactions to humans or animals, because of its anti-inflammatory, antioxidant, antibacterial, anti-cancer and other effects in the world's food, drugs, health products and clinical treatment and other fields have been applied.

## 3 The cause and pathogenesis of OP

OP encompasses a range of bone conditions resulting from diverse factors, where the calcification process in bone tissue remains unaffected, calcium salt to the normal ratio of the matrix, and the unit volume of bone tissue reduction is characterized by metabolic bone disease. In most cases of OP, the loss of bone tissue is primarily caused by increased bone resorption. This disparity in the process of bone breakdown and regeneration leads to an increased susceptibility to experiencing fractures, which can significantly impact the quality of life and increase mortality rates in both men and women (Rinonapoli et al., 2021).

**FIGURE 3:**
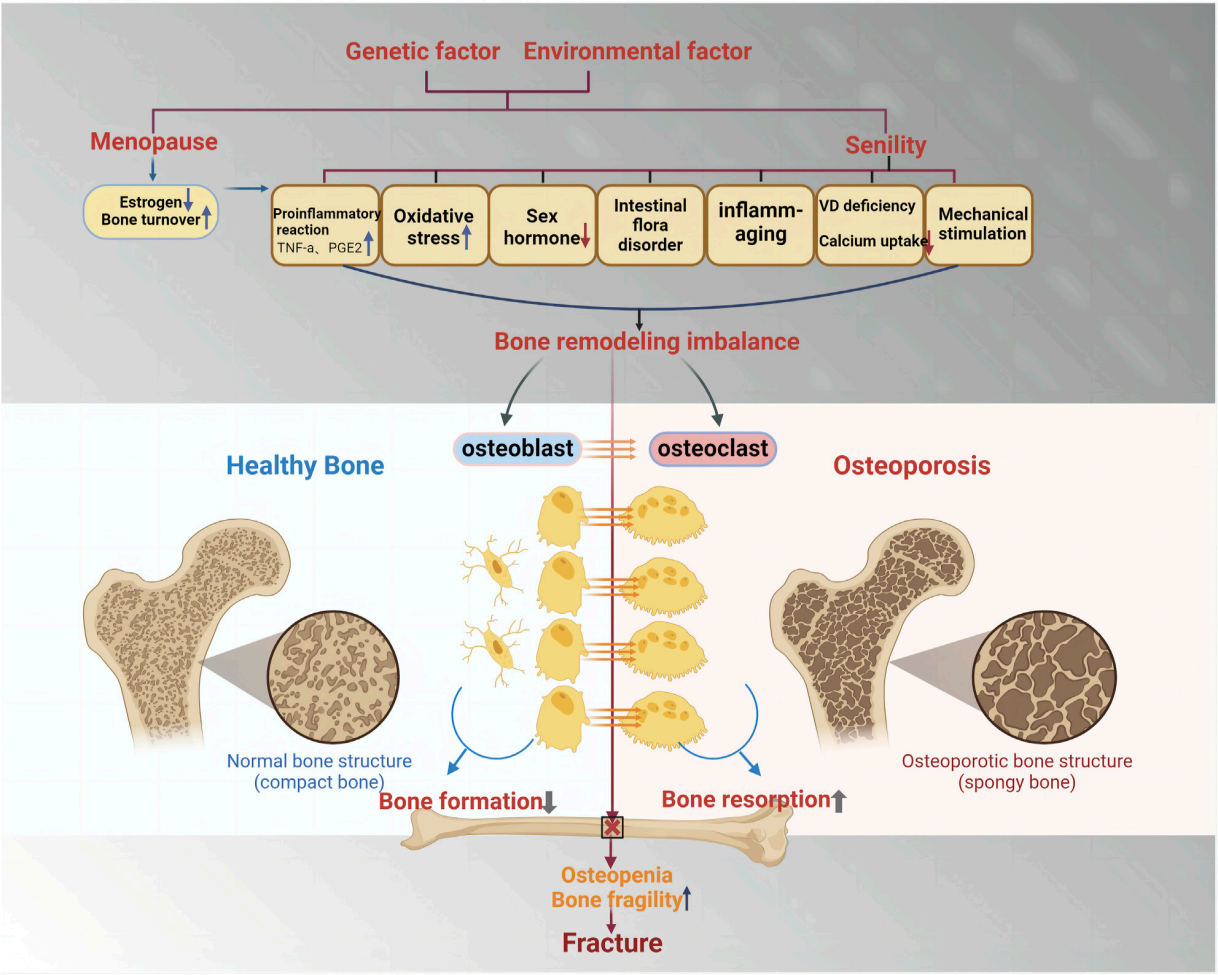
The cause and mechanism of primary osteoporosis. Primary osteoporosis is mainly determined by genetic factors and environmental factors and is divided into two categories: postmenopausal osteoporosis and age-related osteoporosis. Enhanced pro-inflammatory response, oxidative stress, reduced secretion of sex hormones, and intestinal flora disorder will lead to the imbalance of bone reconstruction, the reduction of osteoblast formation, and the increase of osteoclast formation, which will lead to the enhancement of bone absorption and the reduction of bone mass, thus inducing fracture.

The causes of OP can be classified into primary and secondary categories (Figure 3). Based on the established theory, OP is primarily viewed as a bone remodeling disturbance stemming from factors like estrogen insufficiency or the aging process (referred to as primary OP). Conversely, secondary OP arises due to alternative underlying health conditions or medication use that contribute significantly to bone mass depletion (Zhang et al., 2022). Primary OP mainly includes senile and postmenopausal women. Secondary OP includes nutritional, disuse, endocrine, and so on. In women, menopause - the cessation of ovarian function, is one of the main causes of primary OP (Wu D. et al., 2021). The loss of ovarian function is a key factor contributing to bone loss in postmenopausal women, with estrogen believed to play a crucial part in facilitating this procedure (Cheng et al., 2022). Bone remodeling is regulated by estrogen through its control over the synthesis of cytokines and growth factors in bone marrow and bone cells (Crane and Cao, 2014). A number of studies have reported the mechanism of the development of postmenopausal osteoporosis, in which estrogen deficiency in the organism causes inflammatory factors and MicroRNA activation, which leads to disruption of the RANKL-RANK-OPG axis, causing bone loss (Seely et al., 2021). At the same time, estrogen acts as an antioxidant to protect bone against oxidative stress (Shi et al., 2015).

Diabetes is the most common secondary cause of surgery. Several researchers have found an association between the management of blood glucose levels and the likelihood of fractures, as well as increased bone fragility in diabetic patients (Napoli et al., 2017). Studies have shown that people with diabetes have a significantly higher risk of fracture than the general population (Starup-Linde et al., 2017). In a comprehensive Nurses’ Health Study, the incidence of hip fracture in patients diagnosed with T1DM was found to be six times higher than the overall incidence of hip fracture observed in the general population in this particular study, which had an average age of 65 years (Vilaca et al., 2020). Persistent high blood sugar, a hallmark of diabetes, triggers the generation of advanced glycation end-products (AGEs) (Khalid et al., 2022). These molecules can cause significant alterations in the structure of type I collagen and other vital biological components that are central to preserving the structural integrity of bone, consequently impeding the osteogenic process. Research has demonstrated that the receptor for AGEs (RAGE) suppresses osteoblast proliferation by disrupting multiple signaling pathways, such as the PI3 kinase, extracellular signal-regulated kinase (ERK), and Wnt pathways (Zhou et al., 2023). Furthermore, RAGE plays a role in enhancing the development of osteoclasts, which can result in disordered bone formation. The aggregation of AGEs upon their binding to RAGE forms cross-links with bone matrix proteins, which could impact the rigidity and breaking point of the bone (Burr and Allen, 2019). It has been observed that individuals with diabetes often exhibit considerably lower levels of alkaline phosphatase, a critical enzyme for bone mineralization. A reduction in the activity of alkaline phosphatase is typically detected following the diagnosis of diabetes. In consequence, diabetes adversely influences bone metabolism, leading to the weakening of cellular functions and the deterioration of the extracellular matrix. This can manifest in the form of bone loss, an altered bone microarchitecture, a reduction in bone turnover, and an increased susceptibility to fractures even under low-impact conditions (Kemmler and von Stengel, 2019). Additionally, the development of mineral and vitamin D deficiencies can be attributed to gastrointestinal dysfunction, malnutrition, and malabsorption, ultimately leading to deterioration of bone health (Minisola et al., 2021). Notably, patients with chronic liver disease are at a higher risk of fracture due to hepatic osteodystrophy. Clinical conditions associated with wasting osteoporosis include spinal cord injuries as well as other neurological and neuromuscular disorders, post-fracture immobilization, and prolonged bed rest (either actual or simulated) (Ponzano, 2022). This results in immediate bone loss in the trabecular and cortical regions of the bone, a relative increase in bone resorption, and a decrease in bone formation. Thus, disuse is considered to be one of the main factors contributing to rapid bone loss and OP. In addition, alterations in osteoblastic pathways are thought to play a crucial role in the bone loss associated with wasted bone.

Diabetes is the most common secondary cause of OP. Some researchers have found that there exists a relationship between the management of blood sugar levels and the likelihood of experiencing fractures and that people with diabetes have increased bone fragility (Napoli et al., 2017). Studies have shown that people with diabetes have a significantly higher risk of fracture compared to the general population (Wallander et al., 2017). The occurrence of hip fractures among individuals diagnosed “with T1DM in a comprehensive Nurses” Health study was found to be sixfold greater compared to the overall prevalence of hip fractures observed within the general population sample of this particular investigation, who had an average age of 65 years (Ballane et al., 2014). Therefore, bone metabolism is negatively affected by diabetes, leading to impaired cell function and degradation of the extracellular matrix, which leads to bone loss, changes in bone microstructure, reduced bone turnover, and susceptibility to low-trauma fractures (Asadipooya and Uy, 2019). Furthermore, the occurrence of mineral and vitamin D deficiencies can be attributed to gastrointestinal disorders, malnutrition, and malabsorption, ultimately resulting in the deterioration of bone health. It is worth noting that individuals with chronic liver disease face an elevated risk of fractures due to hepatic bone dystrophy (Sobh et al., 2022). Clinical conditions linked to disuse OP encompass spinal cord injury, along with other neurological and neuromuscular disorders, immobilization following fractures, and prolonged periods of bed rest (whether actual or simulated) (Brent et al., 2021). This results in immediate loss of bone in trabeculae and cortical compartments, along with a relative increase in bone resorption and a decrease in bone formation (Seeman, 2003). Hence, disuse is considered one of the primary factors leading to rapid bone loss and OP. Additionally, alterations in osteocyte pathways are believed to play a crucial role in bone loss associated with bone disuse (Rolvien and Amling, 2022).

Initial investigations have indicated that bone loss begins shortly after injury in patients with spinal cord injury and nerve/neuromuscular disorders. During the first year, subregional bone sites may experience bone loss rates of up to 2%–4% per month (Poole et al., 2015). Nevertheless, disuse OP encompasses not only overall systemic bone loss but also localized bone loss in various conditions. For example, in a group of post-stroke patients, the lower limbs impacted by the stroke showed reduced mineral and geometric properties in comparison to the unaffected upper limbs (Kazakia et al., 2014).

From a mechanism point of view, an important factor that causes OP is oxidative stress, which usually refers to an excess of free radicals, an imbalance between free radical oxidants and antioxidants, resulting in cell damage that affects its contents (Manolagas, 2010). In addition, signaling pathways such as NF-κB are activated to produce downstream cytokines. As the oxidizer continues or increases, the entire physiological system is affected. Changes in cells and their organelles alter cell function by inhibiting or activating various cellular pathways (Liu and Tang, 2020).

The bone mineral density seems to be influenced by the consumption of phenols, as they function as scavengers of free radicals and safeguard against oxidative damage to bone cells (Chisari et al., 2019). Polyphenols target a range of molecular mechanisms and signaling pathways, including mTOR, NF-κB, and Wnt/β-catenin. Furthermore, they interact involving intracellular signaling cascades, including PI3K, PKB/Akt, tyrosine kinases, protein kinase C (PKC), and MAPKs (Maleki Dana et al., 2021). To date, a multitude of investigations have confirmed that curcumin plays a pivotal role in modulating the signaling pathways implicated in the pathogenesis of osteoporosis.

## 4 Mechanism of action of CUR against OP: Based on multiple pathways

The Wnt signaling pathway is highly conserved in biological evolution and is essential for core processes such as organism construction, cell proliferation, differentiation, and polarity determination (Habib and Acebrón, 2022). There are 19 Wnt proteins in mammals that differ in sequence but share lipid modifications, secreted glycoproteins, and conserved cysteine residues, features that enable enhanced function via Frizzled receptors and the LGR5/6 complex (Martin-Orozco et al., 2019). Wnt signaling is classified into classical (β-catenin-dependent) and non-classical categories. Aberrant expression of the core transcription factor β-catenin is closely associated with a variety of diseases, especially tumor formation (Wu Z-L. et al., 2021; Zhu et al., 2022). In the Wnt/β-catenin pathway, β-catenin accumulates and enters the nucleus to regulate gene expression. In addition, several components of the Wnt pathway are strongly associated with the development of cancer and degenerative diseases (SMA, 2020). In addition, hereditary bone mass abnormalities are associated with specific mutations in LRP5, Wnt5a, ROR2, and DVL1, and these abnormalities not only affect the skeletal system but also spread to other parts of the body (Table 2). These findings not only reveal the strong connection between the Wnt signaling pathway and human health but also deepen our knowledge of the signaling mechanisms.

**TABLE 2:** Diseases associated with Wnt signaling pathway components.

Disease	Component	Ref.
Bone density defects	LRP5	Gong et al. (2001) Jenkins et al. (2009)
	LGR4	
	WNT1	
	WNT16	
	WTX	
Tooth development defects	LRP6	Jenkins et al. (2009) Lammi et al. (2004)
	WNT10A	
	WNT10B	
	AXIN2	
Robinow syndrome	WNT5A	White et al. (2015); van Amerongen et al. (2012)
	DVL1	
	ROR2	

Relevant components of the Wnt signaling pathway, such as Wnt5a, ROR2, and DVL1, have an impact on degenerative diseases. Especially in metabolic bone diseases, the regulation of the Wnt signaling pathway may lead to abnormal bone metabolism. Abbreviations: LRP5-low density lipoprotein receptor-related proteins 5; LGR4 - leucine-rich repeat-containing G proteincoupled receptor 4 Gene; WTX-Wilms' tumor X; AXIN2-Axis Inhibitor 2.

Furthermore, the Wnt signaling pathway is critical for OP, as it regulates the growth, differentiation, and apoptosis of mesenchymal stem cells (MSCs), driving the differentiation of bone marrow progenitor cells to osteoblasts and influencing bone formation and growth (Amjadi-Moheb and Akhavan-Niaki, 2019). This process is achieved by inhibiting adipocyte differentiation-associated transcription factors and enhancing osteoblast differentiation-associated transcription factor expression, balancing adipogenesis and osteogenesis. In the absence of β-catenin, cells may develop into chondrocytes rather than osteoblasts (Yuan X. et al., 2016). Wnt signaling also promotes osteoblast formation and inhibits anisocytosis, in contrast to PPARγ. In some cases, increased Wnt signaling reduces osteoclast genesis and bone resorption by elevating osteoblast protein expression in osteoblasts and inhibiting the binding of RANKL to osteoclast precursors using bone-protecting aggrecan, which inhibits osteoclast differentiation and activity and reduces bone resorption (Srisubin, 2021).

NF-κB is a key factor in the regulation of immune cells, affecting survival, activation, and differentiation, especially innate immune and inflammatory T cells. It is bound by p50/p65 to IκB in an inactive complex (Baldwin, 1996). signals such as TNF activate IKK, which degrades IκB and releases NF-κB into nuclear regulatory genes (Hayden and Ghosh, 2014). Continued activation may lead to aberrant cell proliferation. NF-κB is important in inflammation, promoting pro-inflammatory factors and COX-2 expression, with potential as an anti-inflammatory and anti-cancer drug. In osteoporosis, NF-κB regulates osteogenic and osteoclast function (Deng et al., 2024). Upon activation, p50/p65 enters the nucleus to promote transcription, especially in the RANKL-RANK environment to induce osteoclast formation, and with c-Fos to form AP-1, which is critical for osteoclast development (Qu et al., 2022). It was also found that inhibition of the NF-κB signaling pathway suppressed RANKL-induced osteoclast differentiation. In addition, the cytokine TNF-α promotes RANK-RANKL binding through activation of the NF-κB pathway, which in turn promotes osteoclast genesis (Amin et al., 2020). The pathogenesis of OP is closely related to inflammation and oxidative stress, in which NF-κB plays a central role, responding to oxidative stress by decreasing SOD expression and increasing MDA synthesis (Neganova et al., 2021). ROS accumulation also enhances NF-κB through phosphorylation activation, leading to the upregulation of inflammatory cytokines and NLRP3 inflammasome synthesis (Sho and Xu, 2019). AP-1, a downstream factor of NF-κB, is responsible for regulating oxidative stress-induced pro-inflammatory cytokine production (Lee et al., 2017). Notably, the NF-κB signaling pathway is regulated by estrogen and affects ERα and β activity (Chen et al., 2018). In menopausal women, estrogen deficiency leads to increased secretion of pro-inflammatory cytokines such as TNF-α and IL-6, which in turn may induce OP (Salamanna et al., 2018).

After a long period of extensive research, the PI3K-AKT pathway is also a compelling area of study due to its multiple functions. This signaling pathway involves key proteins such as PI3K and Akt, which play critical roles in mediating growth factor signaling, organismal growth, and regulation of fundamental cellular processes (He et al., 2021). In addition, the PI3K-AKT pathway affects cell differentiation, metabolic activity, cytoskeletal organization, and key processes such as apoptosis and cancer cell survival (He et al., 2021). Thus, this pathway is intricately linked to a range of health problems such as osteoporosis. Osteoblast growth and differentiation are regulated by PI3K/AKT and osteoblast signaling pathways involved in the OP process (Ponzetti and Rucci, 2021). It was found that reducing p-PI3K and p-AKT expression inhibited PI3K/AKT pathway activation in osteoblasts. In OP model rats, although the mRNA levels of PI3K, PDK1, and Akt were stable, the expression of phosphorylated proteins was significantly decreased (Jantan et al., 2021). LY294002, a PI3K inhibitor, blocked PI3K/Akt signaling in osteoblasts and inhibited cell proliferation, ALP activity, calcium accumulation, and expression of osteogenic markers, indicating that the PI3K/Akt pathway is essential for osteoblast function and mineralized bone formation is critical (Zhou et al., 2019). Also, LY294002 promoted the mRNA expression of Caspase-3 and Caspase-9. Dexamethasone, as a synthetic glucocorticoid, downregulated p-PI3K and p-AKT, upregulated GSK-3β expression, inhibited osteoblast proliferation and induced apoptosis, which was useful for the treatment of glucocorticoid-induced OP (Deng et al., 2019). In contrast, IL-37 activated the PI3K/AKT pathway, promoting bone formation-related gene expression, mineral deposition, and ALP function in MSCs. PI3K/AKT inhibitors partially reversed the enhancing effect of IL-37 on osteogenic differentiation of MSCs (Ye et al., 2019).

Signaling between the cell surface and the nucleus is handled by a group of protein kinases, MAPK, which can be activated by external factors such as hormones, stress, and adhesion, and are key to cellular communication (Jarouliya and Keservani, 2019). MAPK gets its name from its activation by mitogens such as growth factors (Yue and López, 2020). Across the evolutionary spectrum, from yeast to *Homo sapiens*, the MAPK cascade maintains a consistent three-tiered kinase configuration. This includes the presence of upstream kinases like MAP4K and downstream effectors such as MAPKAPK (Rahman, 2018). The pathway is activated by a three-order enzymatic cascade from MAP3K, MAPKK to MAPK, which regulates cell growth, differentiation, stress adaptation, and inflammation. The MAPK signaling pathway includes ERK1/2, JNK, P38, and ERK5, which respond to different stimuli and are involved in a variety of cellular processes (Guo et al., 2020). Factors related to OP such as RANKL, OPG, PTHBMP, TGF-B, IL-1, IL6, TNF-a, and estrogen are associated with the MAPK signaling pathway. RANKL promotes osteoclast differentiation through the activation of RANK, which then activates signaling pathways such as NF-KBNFATc1, AP-1, MAPK, and so on. Estrogen, on the other hand, promotes osteoblast proliferation and inhibits osteoclast apoptosis through activation of the MAPK pathway, such as ERK1/2, P38, and JNK, and plays an anti-OP role (Su et al., 2024).

The therapeutic potential of curcumin for osteoporosis is well-documented and is supported by a substantial body of evidence derived from a variety of biological mechanisms and a considerable number of long-term studies. All of these studies have yielded positive results, and some have progressed to the clinical trial stage. However, there is still a paucity of relevant clinical research data. Consequently, there has been a notable surge in scientific interest in curcumin, largely due to its remarkable capacity to safeguard bone health and its vast range of therapeutic applications. Findings from preclinical and a limited number of clinical studies indicate that curcumin exerts a profound influence on the activities of osteoblasts and osteoclasts. By fostering bone formation while impeding the development of osteoclasts, curcumin plays a pivotal role in promoting bone health (Yang et al., 2023b).

### 4.1 Mechanism of curcumin regulating Wnt pathway in osteoporosis

Turmeric root contains a naturally occurring compound called CUR, whose pharmacological effects have been extensively studied, and curcumin has antioxidant and anti-inflammatory properties (Ahmad et al., 2020). CUR, a bioactive compound, engages in direct interactions with a range of molecular entities such as COX-2, DNA polymerase, lipoxygenase (LOX), glycogen synthase kinase-3β (GSK-3β), and cytokines including tumor necrosis factor-alpha (TNF-α) (Grover et al., 2021). Furthermore, it modulates the activity of various transcription factors in an indirect manner, encompassing NF-κB, activator protein 1 (AP-1), β-cyclin, signal transducers and activators of transcription (STAT), and peroxisome proliferator-activated receptor gamma (PPARγ). The multifaceted regulatory influence of curcumin on these molecular targets underscores its robust anti-inflammatory properties, suggesting its potential as a therapeutic agent in the treatment of inflammation-associated disorders (Chainoglou and Hadjipavlou-Litina, 2019). For instance, many studies have shown that the typical WNT/β-catenin pathway stimulates inflammation and that ROS activates the typical WNT/β-catenin pathway through oxidation and inactivation of nuclear oxyreducing proteins (a redox-sensitive regulator) under NOX stimulation, thereby stimulating the oncogenic process (Vallée et al., 2019a). CUR exerts its influence on the Wnt signaling pathway by reducing the activity of β-catenin, thereby downregulating the expression of genes targeted by β-catenin (Li et al., 2021). Computational studies suggest that curcumin may impede the recruitment of axin to the cell membrane, which is crucial for preserving the integrity of β-catenin destruction complexes in the normal cellular context. This action prevents the accumulation of β-cyclin in the nucleus, thus hindering its interaction with lymphocyte enhancer factor/T-cell-specific transcription factor (Lef/Tcf) complexes and dampening the transcriptional activation of target genes (Hosseinzadeh et al., 2018). Consequently, this leads to the suppression of hepatocellular carcinoma cell proliferation and the induction of programmed cell death, or apoptosis. This shows that curcumin has a significant role in the treatment of inflammation and cancer by regulating the Wnt signaling pathway.

CUR has the potential to affect the Wnt signaling pathway, which is important in the treatment of OP. CUR appears to inhibit this pathway, resulting in an anti-osteoporotic effect on the organism (Yang et al., 2023b). Some researchers found that curcumin gavage inhibited ovxinduced EZH2 mRNA levels in mandible and femur, and ovx-induced upregulation of the number of EZH2-positive cells was reduced (Toyokawa et al., 2019). In contrast, CUR gavage restored ovx-mediated downregulation of β-Catenin and Runx2 mRNA levels (Faienza et al., 2024). The protective influence of curcumin against OP is likely attributed to its capacity to suppress the transcriptional activity of EZH2, which in turn leads to a diminished activation of the Wnt/β-Catenin signaling pathway (Brockmueller et al., 2023). In addition, CUR alters downstream effectors of the Wnt signaling pathway, including c-Myc and cell cycle protein D1 (Vallée et al., 2019b). Deletion of the mouse β-catenin gene was found to promote the differentiation of MSCs to adipocytes, while hindering the differentiation of osteoblasts. This implies that the regulation of bone formation is largely influenced by the Wnt/β-catenin pathway (Song et al., 2012). Chen et al. showed that CUR has the potential to enhance nuclear translocation of β-catenin (Chen et al., 2016b). This was primarily achieved by increasing the enzymatic activity of GSK3β phosphorylated glycogen synthase, resulting in beneficial effects on bone health. Furthermore, it has been observed that the intake of curcumin, at a rate of 100 mg/kg/d, substantially alleviated the decrease in bone mineral density and the loss of bone mineral in rats subjected to conditions that mimic glucocorticoid-induced osteoporosis (Chen et al., 2016b). This result was attributed to the modulation of the Wnt/β-catenin pathway and the alleviation of impaired osteoblast differentiation in the *in vitro* setting. In addition, it has been found that glucocorticoid (GC)-induced Wnt/β-catenin mRNA expression levels were significantly downregulated in OP model rats, whereas Cur intervention increased serum OCN levels and decreased C-terminal peptide of type I collagen (Yang et al., 2023a). The expression of genes pivotal to osteoblast differentiation and function, such as ALP, Runx2, and osterix (Osx), was found to be elevated. ALP and OCN serve as indicators of the bone formation process, in contrast to C-telopeptide of type I collagen, which is a biomarker for bone resorption (Tang et al., 2020). It is evident that CUR can promote bone formation and inhibit bone resorption through the regulation of the Wnt/βcatenin signaling pathway (Chen et al., 2016c).

### 4.2 Regulation of NF-κB signaling pathway by curcumin in osteoporosis

In conclusion, curcumin shows promise in the therapeutic management of osteoporosis (OP) by targeting the regulation of the NF-κB/IL-6 signalling pathway, a mechanism that significantly inhibits inflammatory processes (Yang et al., 2023a). This highlights the critical role of curcumin in alleviating bone health challenges. Additionally, curcumin has been shown to be beneficial in addressing diabetes-induced osteoporosis by positively affecting key signaling pathways such as NF-κB and transforming growth factor β1 (TGF-β1) (Zamanian et al., 2024), which are essential for protecting bone health. Osteoporosis is a common complication in diabetic patients, with a higher prevalence in the diabetic stage. In particular, CUR successfully reversed the overexpression of inflammatory cytokines such as TNF-α, IL-1β, IL-6 and chemokines such as MCP-1 in diabetic samples, which strongly demonstrated its anti-inflammatory efficacy against hyperglycemias-induced lesions (Kong et al., 2021). In a hyperglycemic environment, CUR pretreatment not only promotes differentiation induction of BMSCs, but also significantly enhances angiogenesis (Wang et al., 2012; Zhang et al., 2023), providing a new perspective for the treatment of diabetes-induced osteoporosis. Notably, CUR inhibits the NF-κB pathway, thereby preventing the decline in bone mineral density (BMD) common in diabetic patients (JiaQiang et al., 2021). This discovery has the potential to revolutionize the treatment of osteoporosis. *In vivo* experiments further confirmed that daily CUR treatment at 100 mg/kg effectively prevented bone loss and promoted angiogenesis in diabetes-induced osteoporosis (Yang et al., 2023a). Given the strong link between osteoporosis and oxidative stress, it was found that CUR not only has antioxidant and anti-inflammatory effects but also attenuates oxidative stress and promotes bone formation by modulating the MDA/GSH ratio, as demonstrated by the mouse OVX model (JiaQiang et al., 2021). Low concentration of CUR-protected osteoblasts under oxidative stress conditions, reduced the levels of inflammatory factors such as RANKL and IL-6 and reduced the secretion of inflammatory cytokines such as IL-6 and RANKL by inhibiting the phosphorylation of P65 in the NF-κB signaling pathway, which in turn promoted bone formation (Li et al., 2020b).

In addition, CUR reduced the intranuclear expression of NF-κB p65 by inhibiting the phosphorylation of IκBα and its degradation, thereby inhibiting NF-κB activation, which plays a key role in the inhibition of osteoclast genesis in rheumatoid arthritis patients. A large number of studies have consistently shown that CUR reverses biological abnormalities in inflammatory and oxidative processes by decreasing the activity of NF-κB transcription factors, as well as reducing the expression of p-p65 and the transcription of its phosphorylated factors (Zhong et al., 2016).

In summary, the activity of the NF-κB signaling pathway, a key pathway regulating the expression of IL-6 and RANKL, is tightly regulated by CUR, which in turn affects the osteogenesis process in preosteoblasts. CUR, by virtue of its dual antioxidant and anti-inflammatory effects, promotes bone formation by inhibiting the phosphorylation of P65, and exhibits inhibitory effects on osteoclasts in in vivo experiments, reducing bone resorption, and may have directly promoted osteoblast activity and bone formation. This series of findings highlights the centrality of the NF-κB signaling pathway in the mechanism of action of CUR.

### 4.3 Curcumin interferes with osteoporosis and PI3K-AKT signaling pathway

The PI3K/AKT signaling pathway, as a well-recognized anti-apoptotic and pro-survival signal transduction pathway, is of great importance. Numerous studies have shown that CUR exhibits significant therapeutic potential in the areas of inflammation alleviation, neurological disorders and anti-cancer by upregulating the expression of PI3K and AKT proteins (Hamzehzadeh et al., 2018). Especially, in the study of osteoporosis model rats, although the mRNA expression levels of PI3K, PDK1 and Akt remained stable in bone tissues, the protein expression of their phosphorylated forms was significantly decreased, a finding that reveals key signaling changes during osteoporosis pathology (Wang et al., 2023). Further exploration revealed that when PI3K activation was blocked, the mRNA expression of cell proliferation ability, ALP activity, calcium accumulation, and osteogenesis-related genes such as OCN, Osterix, and Runx2 were suppressed, whereas the mRNA expression of apoptosis-related genes, Caspase-3 and Caspase-9, increased accordingly, highlighting the central role of the PI3K/AKT pathway in the central role of the PI3K/AKT pathway in maintaining bone health (Aimaiti et al., 2020). It is particularly exciting that curcumin was shown in another study to be able to elevate the protein and mRNA levels of ALP, COL1 and RUNX2, and activate the PI3K/AKT/Nrf2 signaling pathway, which effectively promotes the osteogenic differentiation of human periodontal stem cells, providing a new strategy for bone regeneration therapy (Xiong et al., 2020). In addition, CUR has demonstrated its unique efficacy in the treatment of osteoarthritis by precisely intervening in the PI3K-AKT signaling pathway, which protects joint health by inhibiting the PI3K/AKT/mTOR signaling cascade, promoting autophagic response, reducing joint inflammation and restoring joint homeostasis (Song et al., 2022). Although activation of the PI3K/AKT/mTOR pathway has a positive effect on chondrocyte proliferation and differentiation and reduces apoptosis, there is a dearth of research on the specific mechanisms and effects of curcumin in treating OP through this pathway. However, there have been preliminary studies such as the work of Riva et al. By administering high-dose curcumin (1000 mg/day) orally to 57 healthy subjects with low BMD for a period of 24 weeks, not only did the subjects confirm good tolerance of CUR, but also positive changes in BMD were observed (Riva et al., 2017). In response to the current status of OP as a common complication of spinal cord injury and the lack of treatment options, the Hatefi team’s study was equally encouraging. They found that 6 months of CUR supplementation (at a dose of 110 mg/kg) significantly elevated BMD parameters and effectively reduced biomarker levels of bone loss in spinal cord injury patients, providing strong evidence for slowing the process of OP (Kheiridoost et al., 2022). In summary, CUR, as an active ingredient in natural herbs, has shown the first signs of its ability to treat OP by modulating signaling pathways. Although the specific mechanism and data on curcumin’s treatment of osteoporosis through the PI3K pathway are yet to be enriched, the potential it exhibits undoubtedly opens up a broad prospect for future in-depth research and clinical application.

### 4.4 Curcumin interferes with osteoporosis and MAPK signaling pathway

To date, various experiments on the effects of curcumin on p38 MAPKs and related diseases have been successively conducted (Peng et al., 2021b). Some researchers found that curcumin induced p38 MAPK phosphorylation, which increased apoptosis and facilitated tumour suppression in retinoblastoma cells (Shehzad and Lee, 2013). Curcumin positively affects glucose uptake in L6 myotubular cells through activation of p38 MAPKs (Ferraro et al., 2014). Curcumin inhibits COX-2 expression by suppressing p38 MAPK activation in human keratinocytes. In ovarian cancer, curcumin upregulated the phosphorylation of p38 MAPK, thereby promoting apoptosis (Wu et al., 2022). The role of curcumin in reducing inflammation by inhibiting the MAPK pathway has been validated in a variety of models and experiments. In a study of middle cerebral artery occlusion in rats, we found that curcumin reduces inflammation by inhibiting the TLR4/p38/MAPK pathway (Huang et al., 2018). In a separate investigation, mice were subjected to colitis through the administration of dinitrobenzene sulfonic acid. Within this context, curcumin demonstrated its efficacy by suppressing the activation of the p38 MAPK and NF-κB signaling cascades, which resulted in a noticeable decrease in the levels of the pro-inflammatory cytokine interleukin-1 beta (IL-1β). Furthermore, curcumin’s anti-inflammatory properties were also evident in its capacity to mitigate hepatic inflammation and cell death (Ma et al., 2023). This was achieved through the modulation of inflammatory signaling pathways, notably the p38 MAPK pathway, consequently diminishing the expression of cytokines implicated in inflammation, such as IL-1β (Ashrafizadeh et al., 2020). In an experimental asthmatic condition replicated in mice, curcumin exerts regulatory effects on secretory phospholipase A2 function, consequently leading to a diminution in the concentrations of the pro-inflammatory mediators Cox-2 and Prostaglandin D2 (Memarzia et al., 2022). CUR and its nano formulation have demonstrated the ability to mitigate and potentially avert the process of programmed cell death by impeding the signaling pathways of Toll-like receptor 4 (TLR4) and NF-κB, as well as MAPK cascade. In a rodent model of periodontal disease, the topical application of curcumin in nanoparticle form has been observed to curtail inflammatory responses by inhibiting the initiation of NF-κB and p38 MAPK pathways (Zambrano et al., 2018). In human intestinal microvascular endothelial cells exposed to vascular endothelial growth factor, the presence of curcumin has been shown to curtail the process of angiogenesis. This effect is achieved through the suppression of COX-2 expression at both the transcriptional and translational levels, thereby hindering the synthesis of prostaglandin E2. Additionally, curcumin exerts its anti-angiogenic influence by impeding the activation of the JNK and p38 MAPK signaling pathways (Saberi-Karimian et al., 2019). In the spectrum of inflammatory responses observed in inflammatory bowel disease, the engagement of endothelial cell adhesion molecules stands out as a crucial component. Studies have confirmed that curcumin inhibits vascular cell adhesion molecule expression by affecting Akt, NF-κB, and p38 MAPK, and reduces TNF-α, IL-1β, and lipopolysaccharides, ultimately leading to a reduction in inflammation (Patel et al., 2020).

**FIGURE 4:**
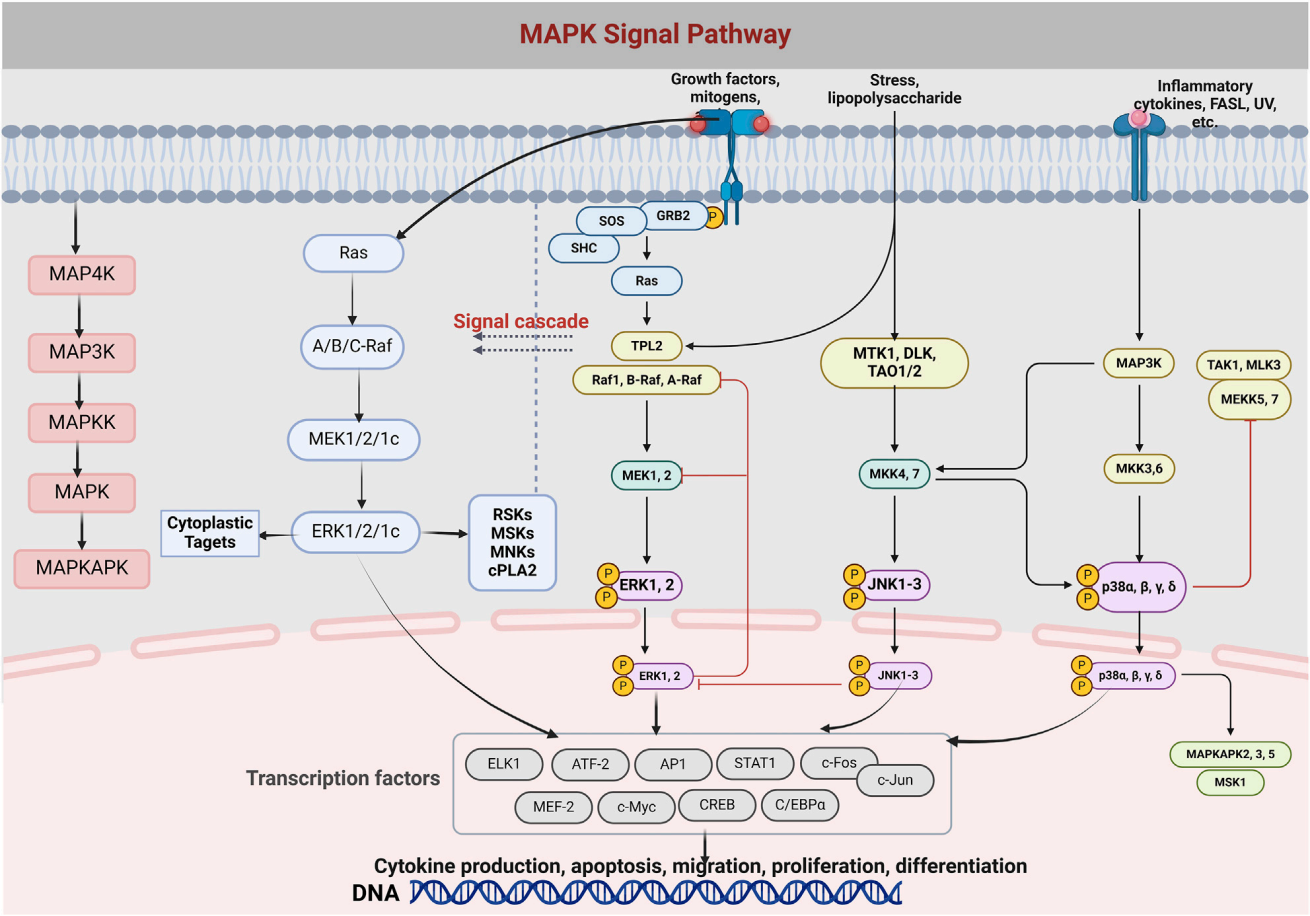
The diagram depicts the formation and differentiation of the MAPK/Erk signaling pathway and the cascade of MAPK. MAPK, mitogen-activated protein excitation, MAPK exists in the cytoplasm and can be translocated to the nucleus to catalyze the phosphorylation of dozens of cytoplasmic proteins and many nuclear transcription factors.

While inflammation and oxidative stress are the keys to osteoporosis, based on the above mechanism of action of curcumin mediating the MAPK pathway to reduce inflammation and anti-oxidative stress to treat various diseases, it can be surmised that curcumin based on the MAPK pathway to treat OP also has similar targets and mechanisms. It turns out that this is indeed the case. A comprehensive study showed that taking CUR significantly increased BMD and alleviated bone loss in postmenopausal women with OP. In a 12-month clinical trial that was randomized and double-blinded, involving 60 postmenopausal women, the concurrent administration of curcumin at a dosage of 110 mg daily and alendronate at 5 mg daily was observed to notably enhance BMD and more effectively diminish the levels of bone resorption markers compared to the use of either drug alone (Khanizadeh et al., 2018). The potential synergistic effect of CUR and alendronate indicates a promising approach for both the amelioration and prevention of OP in postmenopausal women. Furthermore, a study by Khanizadeh and colleagues, involving a 6-month randomized, triple-blind trial with 120 participants, demonstrated that the concurrent application of nano cellular curcumin integrated with black seed oil notably decreased serum levels of bone turnover biomarkers. These markers include ALP, osteocalcin, and bridging proteins, thereby potentially mitigating the risk of osteoporosis among this demographic (Kim et al., 2011). Several studies have shown that CUR positively affects the P38 MAPK signaling pathway, resulting in anti-inflammatory, neuroprotective and apoptotic effects (Shamsnia et al., 2023). Curcumin has demonstrated the ability to decrease the phosphorylation of p38 MAPK, a mechanism that positions it as a promising therapeutic agent for osteoporosis management, and a study conducted by Chen and colleagues demonstrated that CUR has a protective effect on osteoblasts, preventing DeX-induced apoptosis. This was achieved by inhibiting the expression of pro-apoptotic proteins and enhancing the ERK pathway (Figure 4) (Chen et al., 2016d). These findings suggest that CUR is an effective drug for the treatment of glucocorticoid-induced OP. Heo et al. also found that CUR was able to impede the differentiation of BMMs to osteoblasts by inhibiting RANKL-induced expression of the osteoclast transcription factors c-Fos and NFAT1 (Heo et al., 2014). In addition, it activated the downstream MAPK pathway (Yang et al., 2023a). The findings indicated that the *in vitro* application of curcumin in a mouse model led to a significant suppression of osteoclast genesis and the maturation of osteoclasts. Consequently, curcumin has the potential to be utilized in the therapeutic management of osteoporosis by modulating the MAPK signaling pathway.

## 5 Conclusion and perspectives

In recent years, a comprehensive review of various signaling pathways implicated in OP, when integrated with the therapeutic mechanisms of curcumin, has revealed its multifaceted role in bone health. CUR demonstrates the capacity to mitigate inflammation within bone tissues, curb the differentiation and proliferation of osteoclasts, foster the growth of osteoblasts, and reduce oxidative stress. This is achieved through a coordinated regulation of multiple signaling pathways, including but not limited to NF-κB, Wnt/β-catenin, PI3K/Akt, and MAPK. Our study and summary provide evidence and reference for the treatment of OP through multiple signaling pathways mediated by CUR, demonstrating a mechanism characterized by multi-target, multi-pathway and multi-level interactions. Although relevant studies have confirmed that CUR, as a component of Chinese herbal medicine, can treat OP by regulating related proteins and has received much attention and made some progress in recent years, there are still many challenges and problems that need to be solved. One of the main challenges is the low bioavailability of curcumin, which leads to a decrease in the therapeutic effect of the compound. Therefore, researchers are trying to improve the clinical application of curcumin through nanotechnology. In addition, although some controlled *in vitro* experiments have demonstrated the potential of curcumin in the treatment of osteoporosis, these experiments have not clearly demonstrated that the effects of the compound on bone-forming and bone-resorbing cells can be manifested in actual bone tissue. Moreover, research on the mechanism of action of curcumin in the treatment of osteoporosis is still predominantly confined to preclinical animal and cellular experiments, which lack sufficient clinical data support. Consequently, it is imperative to conduct more high-quality clinical studies in the future to provide further evidence that curcumin can effectively treat osteoporosis through multiple signaling pathways. Finally, curcumin has therapeutic effects on osteoporosis due to its anti-inflammatory effects, but its specific anti-inflammatory mechanisms and targets of action have not been fully clarified as well as studies on related signaling pathways are limited. Therefore, further studies are needed to investigate the common mechanism of action and targets of CUR and other polyphenols based on osteoporosis-related signaling pathways. This will better utilize the advantages of multi-targets, multi-levels and multi-pathways of natural products and provide assistance for more in-depth related studies.
